# Thrombus aspiration and *in situ* thrombolysis via a 6F Guidezilla catheter in elderly patients with high-risk pulmonary embolism: a case series of six patients

**DOI:** 10.3389/fcvm.2026.1877831

**Published:** 2026-08-06

**Authors:** Peng Li, Chengyu Wang, Chu Guo, Yangyang Ji, Yuan Li, Huaisheng Ding

**Affiliations:** 1 West China Hospital Sichuan University, Meishan Hospital, Meishan, China; 2 Department of Cardiovascular Medicine, Meishan People’s Hospital, Meishan, China

**Keywords:** case series, catheter-directed thrombolysis, elderly, Guidezilla catheter, pulmonary embolism, thrombus aspiration

## Abstract

**Background:**

Systemic thrombolysis is often not an option for elderly patients with PE because of bleeding risk. Large-bore catheters (16F−24F) can injure fragile vessels. The 6F Guidezilla guide extension catheter, already used in coronary cases, offers a smaller-caliber choice for thrombus aspiration and local thrombolysis. We report six elderly patients with high-risk PE treated with this approach.

**Methods:**

Six consecutive patients with intermediate-high or high-risk PE underwent thrombus aspiration and *in situ* thrombolysis through a 6F Guidezilla catheter (March 2024 to January 2026). Agents used: recombinant human prourokinase 15–30 mg or urokinase 2 million IU. All had contraindications to systemic thrombolysis. We collected baseline data, procedural metrics, Miller scores (pre/post), SpO₂, hemodynamics, complications, hospital stay, and 30-day outcomes.

**Results:**

The mean age of the patients was 75.8 ± 4.6 years (range 70–81 years), with five patients aged ≥70 years. One patient presented with high-risk PE (shock), and the remaining five were classified as intermediate-high risk. The mean simplified Pulmonary Embolism Severity Index (sPESI) score was 2.7 ± 1.2. The mean Miller score decreased significantly from 18.0 ± 6.7 pre-procedure to 5.2 ± 3.6 post-procedure (*P* < 0.05), with a mean clot clearance rate of 74.1% ± 15.2%. SpO₂ improved from 84.5% ± 5.0% pre-procedure to 97.2% ± 0.8% at 24 h post-procedure (*P* < 0.01). The high-risk patient was successfully weaned off vasopressors within 24 h of the procedure. No major bleeding or procedure-related deaths occurred during hospitalization. The mean hospital stay was 7.8 ± 4.4 days, and no recurrent PE or death was observed within 30 days of follow-up.

**Conclusion:**

The 6F Guidezilla catheter appears safe and effective for thrombus aspiration and *in situ* thrombolysis in elderly PE patients who cannot receive systemic thrombolysis. It achieves good clot clearance, quick clinical recovery, and few complications. When large-bore catheters are unavailable or too risky for elderly vessels, this technique is a practical alternative.

## Introduction

Pulmonary embolism (PE) is a life-threatening event caused by clot obstruction of the pulmonary arteries. Among intermediate-high risk patients, mortality is high even with normal blood pressure. Removing the clot quickly and effectively is critical (1).

The 2019 ESC guidelines recommend anticoagulation as first-line treatment for intermediate-high risk PE (1). But patients can still decompensate despite initial stability, so close monitoring is advised. If hemodynamics worsen, rescue reperfusion should be considered promptly (1).

Intermediate-high risk PE covers a wide spectrum. Some patients have clear danger signs: syncope predicts larger clots, worse RV function, and higher 30-day mortality (6, 7). Severe hypoxemia on arrival predicts in-hospital death (4, 5). Those with ongoing dyspnea and tachycardia despite anticoagulation may need more aggressive intervention (8).

Dedicated PE catheters like the Inari FlowTriever and Penumbra Lightning are large-bore (16F−24F) (10, 11). They remove clots well but can damage vessels, especially in elderly patients. Age-related changes—atherosclerosis, loss of elasticity—make access-site complications and vessel injury more likely.

The 6F Guidezilla™ guide extension catheter was originally made for coronary work but offers a smaller-bore option here. Its inner lumen is wide enough for manual aspiration and for delivering thrombolytics directly into the clot. At 6F it is the same size as a standard diagnostic catheter, which should lower the risk in elderly patients with fragile vessels.

We first tried this in a 70-year-old woman with high-risk PE who could not get systemic thrombolysis because of recent surgery. She did well after thrombus aspiration and *in situ* thrombolysis through the Guidezilla. That case was published in 2024 (12).

Since then we treated five more patients the same way, for a total of six. Most were over 70; the oldest was 81. sPESI scores ranged 1–4, consistent with intermediate-high to high risk. One had syncope; all had hypoxemia, dyspnea, and fatigue—features tied to worse outcomes (4, 6, 8).

We do not have large-bore PE catheters at our hospital. Even if we did, the large size would raise vascular risk in these elderly patients. So we turned to the 6F Guidezilla—it is already in our lab, has enough lumen for aspiration, and works with standard technique.

We present our experience with the 6F Guidezilla in six elderly intermediate-high or high-risk PE patients. Our aim is to show that this small catheter can clear clots effectively and improve clinical status safely in this tough group. We also want to point out that any cath lab with standard equipment can do this—no need for specialized PE devices.

## Materials and methods

### Study design and patients

This was a single-center, retrospective case series conducted at the Department of Cardiovascular Medicine, The Meishan People's Hospital, Meishan, China. We reviewed consecutive patients with acute PE who underwent catheter-directed thrombus aspiration and *in situ* thrombolysis using a 6F Guidezilla™ guide extension catheter between March 2024 and January 2026.

Inclusion criteria were: (1) diagnosis of high-risk or intermediate-high risk PE according to the 2019 ESC guidelines (1); (2) contraindications to systemic thrombolysis (e.g., recent surgery, high bleeding risk); (3) treatment with the 6F Guidezilla catheter for thrombus aspiration and *in situ* thrombolysis; and (4) availability of complete pre- and post-procedure pulmonary angiograms. Patients were excluded if they received systemic thrombolysis or anticoagulation alone without catheter intervention, or if essential clinical or angiographic data were missing.

The study was approved by the Ethics Committee of The Meishan People's Hospital (Ethics Approval No: MY-IBR20260021). Written informed consent was obtained from all patients or their legal representatives before the procedure. The consent forms included permission to use anonymized clinical data and angiographic images for research and publication. All patient data were anonymized by removing personally identifiable information to protect patient privacy. The ethics approval was granted on April 10, 2026.

### Procedure

All procedures were performed in the cardiac catheterization laboratory by the same team of interventional cardiologists with extensive experience in coronary interventions using the Guidezilla catheter. This experience was adapted to the treatment of PE patients.

Step 1: Vascular access and initial angiography. A 6F introducer sheath was inserted into the right femoral vein using the Seldinger technique. Under fluoroscopic guidance, a 0.035-inch guidewire was advanced through a JR 3.5 or 4.0 diagnostic catheter (Judkins Right) from the right atrium, across the tricuspid valve into the right ventricle, and finally into the pulmonary artery. The diagnostic catheter was then exchanged for a 6F pigtail catheter over the guidewire. Pulmonary angiography was performed with iodinated contrast medium to confirm the diagnosis, localize the thrombi, and assess thrombus burden. Images were obtained in multiple projections to fully visualize both pulmonary arterial trees.

Step 2: Guiding catheter placement and wire crossing. The pigtail catheter was removed, and a right ventricular guiding catheter (e.g., Judkins Right or multipurpose catheter) was advanced over the guidewire into the pulmonary artery. A 0.014-inch PTCA guidewire was then advanced through the guiding catheter, carefully crossing the thrombus to reach the distal segment of the affected pulmonary artery.

Step 3: Guidezilla advancement and thrombus aspiration. A 6F Guidezilla™ guide extension catheter (Boston Scientific, Marlborough, MA, USA) was advanced over the PTCA guidewire to the thrombus site. For aspiration, a coronary pressure pump was connected to the Guidezilla catheter via a three-way stopcock. Negative pressure was applied by fully opening the stopcock and pulling back the pump handle. The catheter was then slowly withdrawn while maintaining negative pressure. This aspiration step was repeated 2–3 times until no additional thrombus material could be retrieved.

Step 4: *in situ* thrombolysis. After thrombus aspiration, the Guidezilla catheter was removed and a 6F pigtail catheter was re-advanced over the guidewire. Pulmonary angiography was repeated to assess residual thrombus burden. If significant thrombus remained, the pigtail catheter was exchanged for a guiding catheter, and a 0.014-inch PTCA guidewire was re-advanced through the residual thrombus. The 6F Guidezilla catheter was then re-introduced over the wire to the target site. Recombinant human prourokinase (15–30 mg) was injected slowly through the Guidezilla catheter directly into the residual thrombus over 3–5 min. The dose was divided between the two pulmonary arteries when bilateral thrombi were present. No continuous infusion was used. (Note: In Patient 6, urokinase 2 million IU was used instead of prourokinase, consistent with our previously reported case 12).

Step 5: Post-procedure angiography and closure. The Guidezilla catheter was removed, and a 6F pigtail catheter was re-advanced over the guidewire. Pulmonary angiography was repeated using the same projections as the initial study to assess the effectiveness of aspiration and thrombolysis. The introducer sheath was then removed immediately, and manual compression was applied to the access site for 10–15 min to achieve hemostasis. Patients were transferred to the intensive care unit or cardiology ward for close monitoring. Anticoagulation with unfractionated heparin or low molecular weight heparin was initiated 6 h after the procedure, followed by oral anticoagulation as clinically indicated.

### Data collection

Data were extracted from electronic medical records, procedure reports, and angiographic images using a standardized case report form. All data were reviewed and verified by two independent investigators to ensure accuracy.

Baseline characteristics included age, sex, sPESI score, risk level (high or intermediate-high), comorbidities/triggers (e.g., recent surgery, venous thrombosis, malignancy), and contraindications to systemic thrombolysis. Bleeding risk was assessed qualitatively based on age, comorbidities, and recent surgical history; quantitative tools such as the HAS-BLED score were not routinely available in the retrospective dataset.

**Pre-procedure data** included oxygen saturation (SpO₂) measured by pulse oximetry under supplemental oxygen, heart rate, blood pressure, vasopressor use, B-type natriuretic peptide (BNP) levels (if measured within 24 h before the procedure), and the Miller score calculated from the initial pulmonary angiogram.

Intra-procedure data included total procedure time (from sheath insertion to removal), thrombolytic agent and dose, and any immediate complications (e.g., arrhythmia, vessel injury, hemodynamic collapse).

**Post-procedure data** included immediate SpO₂ after the procedure (before leaving the catheterization laboratory), SpO₂ at 24 h post-procedure, blood pressure and heart rate at 24 h, vasopressor use at 24 h (discontinued, reduced, or unchanged), BNP at 24 h (if measured), and the Miller score calculated from the post-procedure angiogram.

**In-hospital outcomes** included complications (access site bleeding, hematoma, hemoptysis, pulmonary artery injury, death) and length of hospital stay.

**Follow-up** was conducted by telephone interview or outpatient clinic visit at 30 days to record major adverse cardiovascular events (MACE), defined as recurrent PE, rehospitalization for cardiovascular causes, or death from any cause.

### Miller score assessment

The Miller score was used to quantify thrombus burden on pulmonary angiography (13). We selected the Miller score over more recent systems (e.g., Qanadli or Mastora scores) because it remains one of the most widely referenced scoring systems in catheter-directed PE studies, allowing direct comparison with historical benchmarks. This validated scoring system is widely used in PE research (13). The pulmonary arterial tree was divided into 16 segments (9 for the right lung, 7 for the left lung). Each segment was scored as 0 (no thrombus, normal filling), 1 (subtotal occlusion, partial filling defect), or 2 (total occlusion, no contrast filling). The total score ranged from 0 to 32, with higher scores indicating greater thrombus burden.

Two interventional cardiologists, blinded to clinical data and to each other's assessments, independently reviewed all angiographic images and calculated Miller scores. Disagreements were resolved by consensus, with a third reviewer available if needed. The final Miller score for each patient was the consensus score. Clot clearance rate was calculated as [(pre-Miller score - post-Miller score)/pre-Miller score] × 100%.

### Statistical analysis

Statistical analysis was performed using SPSS version 26.0 (IBM Corp., Armonk, NY, USA). Descriptive statistics were used to summarize baseline characteristics and procedural data. Continuous variables were expressed as mean ± standard deviation or median with range, depending on data distribution (BNP levels were non-normally distributed and expressed as median [interquartile range]). Categorical variables were expressed as numbers and percentages.

For pre- and post-procedure comparisons, the Wilcoxon signed-rank test was used for paired non-parametric data due to the small sample size (*n* = 6). A *P*-value < 0.05 was considered statistically significant. No sample size calculation was performed, as this was a retrospective case series including all eligible patients during the study period.

## Results

### Patient characteristics

A total of six patients with acute pulmonary embolism who underwent catheter-directed thrombus aspiration and *in situ* thrombolysis using a 6F Guidezilla catheter were included in this case series. The mean age was 75.8 ± 4.6 years (range 70–81 years), and four patients (66.7%) were female. According to the 2019 ESC criteria, five patients were classified as intermediate-high risk and one patient (Patient 6) as high risk due to hemodynamic instability at presentation (1). The mean sPESI score was 2.7 ± 1.2 (range 1–4).

Comorbidities and triggers included venous thrombosis (*n* = 3), post-operative state (*n* = 2), tumor (*n* = 1), and long-term olanzapine (50 mg nightly) for schizophrenia (*n* = 1). All patients had contraindications to systemic thrombolysis: recent surgery (*n* = 2) and high bleeding risk due to age, comorbidities, or other factors (*n* = 4). Baseline characteristics are summarized in Table 1.

**Table 1:** Baseline characteristics of 6 patients with intermediate-high or high-risk pulmonary embolism.

Patient	Age (years)	Sex	sPESI score	Risk level	Comorbidities/triggers	Pre-procedure SpO₂ (%)	Pre-procedure heart rate (bpm)	Pre-procedure BNP (pg/mL)	Pre-procedure Miller score
Patient 1	73	Male	4	Intermediate-high	Tumor	80	116	349	13
Patient 2	77	Female	1	Intermediate-high	Venous thrombosis	91	94	553	16
Patient 3	81	Female	2	Intermediate-high	Venous thrombosis	90	79	143	18
Patient 4	81	Female	2	Intermediate-high	Venous thrombosis	85	114	179.36	11
Patient 5	73	Male	3	Intermediate-high	Schizophrenia (long-term olanzapine 50 mg)	80	118	19.7	20
Patient 6	70	Female	4	High	Post-operative (varicose vein surgery), bedridden	81	110	50.34	30
Mean ± SD	75.8 ± 4.6	-	2.7 ± 1.2	-	-	84.5 ± 5.0	105.2 ± 15.4	215.7 ± 201.9	18.0 ± 6.7

sPESI, simplified pulmonary embolism severity index; BNP, B-type natriuretic peptide; SD, standard deviation.

### Procedural data

All procedures were successfully completed without intra-procedural complications. Mean procedure time was 92.8 ± 19.6 min (range 75–125 min). Five patients received recombinant human prourokinase (15–30 mg), and one patient (Patient 6) received urokinase 2 million IU (as previously described 12). Balloon predilation was performed in two cases where the thrombus completely occluded the main pulmonary artery branches.

### Thrombus burden reduction

**Figure 1:**
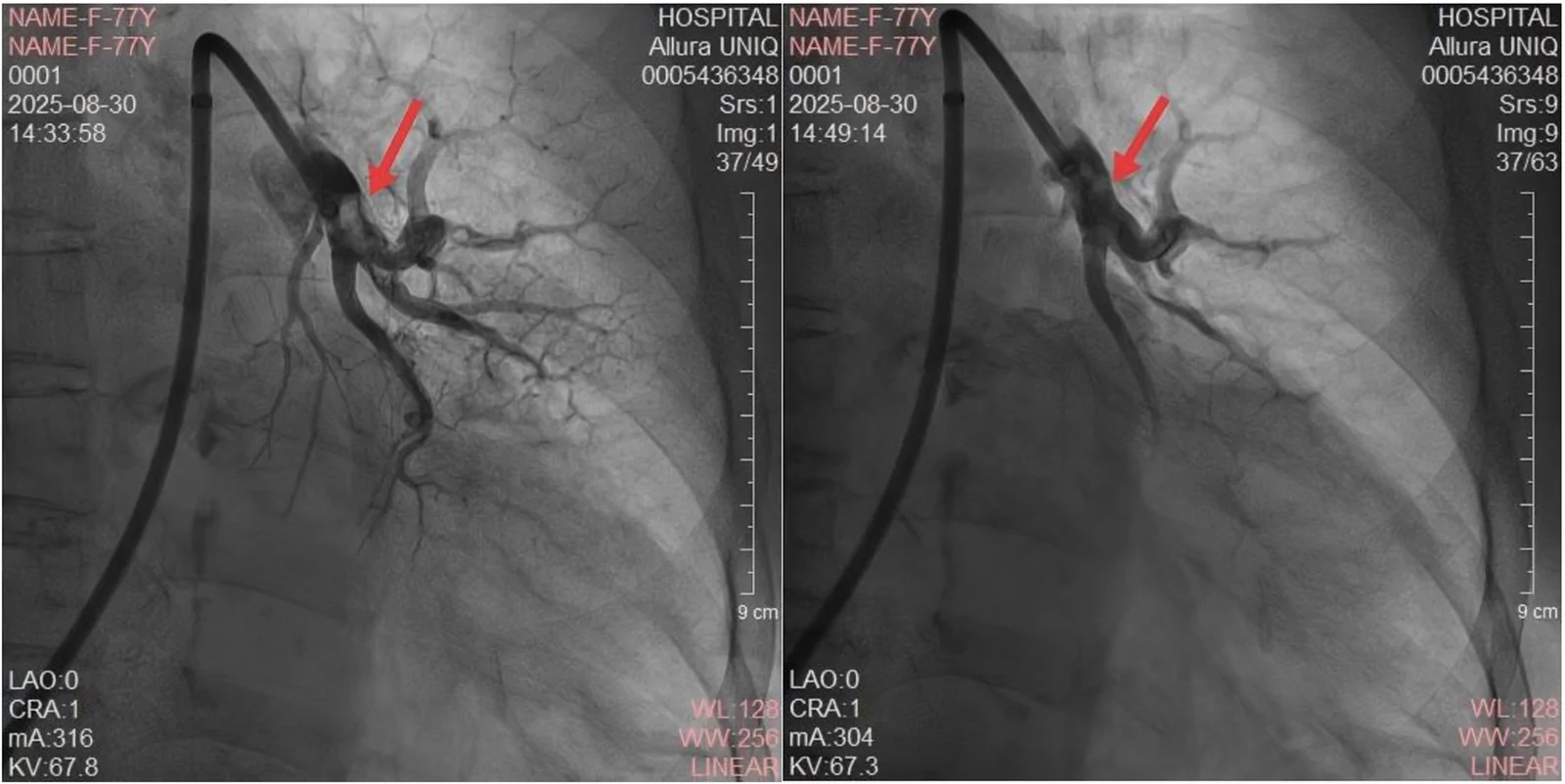
Comparison of left upper pulmonary artery angiography before and after the procedure in patient 2 (a 77-year-old female with intermediate-high risk PE). Pre-procedure Miller score: 16; post-procedure Miller score: 6; clot clearance rate: 62.5%.

All patients showed marked reduction in thrombus burden on post-procedure pulmonary angiography. Mean Miller score decreased from 18.0 ± 6.7 pre-procedure to 5.2 ± 3.6 post-procedure (*P* < 0.05), with a mean reduction of 12.8 ± 3.8. The mean clot clearance rate was 74.1% ± 15.2% (range 61.1%–100.0%). Individual patient Miller scores and clearance rates are shown in Table 2. Figure 1 shows representative pulmonary angiographic images from Patient 2 (a 77-year-old female with intermediate-high risk PE) demonstrating the reduction in thrombus burden after the procedure (pre-Miller: 16; post-Miller: 6; clearance rate: 62.5%).

**Table 2:** Procedural and efficacy outcomes.

Patient	Procedure time (min)	Thrombolytic agent and dose	Immediate post-procedure SpO₂ (%)	24 h post-procedure SpO₂ (%)	Post-procedure BNP (pg/mL)	Post-procedure Miller score	Miller score reduction	Clot clearance rate (%)*	Hospital stay (days)	Complications
Patient 1	105	Prourokinase 20 mg	95	97	58	2	11	84.6	16	None
Patient 2	80	Prourokinase 20 mg	96	97	Not measured	6	10	62.5	7	None
Patient 3	125	Prourokinase 15 mg	93	96	161.64	7	11	61.1	7	None
Patient 4	95	Prourokinase 15 mg	98	98	118.61	0	11	100.0	9	None
Patient 5	75	Prourokinase 30 mg	98	98	Not measured	6	14	70.0	4	None
Patient 6	77	Urokinase 2 million IU	98	99	Not measured	10	20	66.7	4	None
Mean ± SD	92.8 ± 19.6	-	96.3 ± 2.1	97.5 ± 1.0	-	5.2 ± 3.6	12.8 ± 3.8	74.1% ± 15.2%	7.8 ± 4.4	-

* Clot clearance rate = [(pre-Miller − post-Miller)/pre-Miller] × 100%.

SD, standard deviation.

## Clinical improvement

### Oxygenation

All patients showed rapid improvement in oxygenation after the procedure. Mean SpO₂ increased from 84.5% ± 5.0% pre-procedure (under supplemental oxygen) to 95.8% ± 2.0% immediately post-procedure, and further to 97.2% ± 0.8% at 24 h (*P* < 0.01 for pre- vs. 24 h post-procedure). This improvement was sustained throughout hospitalization, with no patients requiring prolonged supplemental oxygen.

### Hemodynamics and vasopressor use

Among the five patients who were hemodynamically stable at presentation (intermediate-high risk), none required vasopressors before or after the procedure. The single high-risk patient (Patient 6) presented with shock requiring norepinephrine (0.4 μg/kg/min). After the procedure, vasopressor requirements were significantly reduced, and the patient was weaned off within 24 h, indicating rapid hemodynamic recovery.

### BNP levels

BNP data were available for all six patients, with a median (interquartile range) of 161.18 (50.34–349.00) pg/mL (mean 215.7 ± 201.9 pg/mL). BNP was measured post-procedure in three patients (Patients 1, 3, and 4), and all showed a decrease (from 349 to 58 pg/mL, 143 to 161.64 pg/mL, and 179.36 to 118.61 pg/mL, respectively). Due to the retrospective nature of the study, post-procedure BNP was not routinely measured in all patients.

## Safety and follow-up

### In-hospital complications

No major bleeding, pulmonary artery injury, or procedure-related death occurred during hospitalization. No hemoptysis or access-site bleeding requiring intervention was observed, confirming the safety of the 6F Guidezilla catheter in this elderly population.

### Hospital stay

The mean length of hospital stay was 7.8 ± 4.4 days (range 4–16 days). Patient 6, who presented with high-risk PE, had a hospital stay of 4 days, consistent with her rapid clinical recovery.

### 30-day follow-up

All patients completed 30-day follow-up. No recurrent pulmonary embolism, rehospitalization for cardiovascular causes, or death occurred within 30 days, indicating favorable short-term outcomes.

## Discussion

We used a 6F Guidezilla™ guide extension catheter for thrombus aspiration and *in situ* thrombolysis in six elderly patients with intermediate-high or high-risk PE. All had contraindications to systemic thrombolysis; five were over 70 (two over 80). Mean clot clearance was 74.1%. Oxygenation and hemodynamics improved quickly. No major bleeding. The Guidezilla appears to work as well as large-bore devices while likely being safer in elderly patients with fragile vessels.

A smaller catheter should mean lower procedural risk in the elderly. Dedicated PE catheters are big—16F to 24F (10, 11). They work but come with real vascular injury risk, especially in older patients with fragile vessels (14, 15). Age stiffens and weakens vessels, raising the odds of access-site problems, dissection, and bleeding (1, 16). In our series, the two 81-year-old patients both tolerated the procedure with no access-site issues.

The 6F Guidezilla was designed for coronary cases (17) and has the same outer diameter as a standard diagnostic catheter. None of our patients had access-site bleeding, hematoma needing intervention, or detectable vessel injury. Going smaller likely cuts down on vascular trauma in elderly patients.

Large-bore complications are real. According to the FDA MAUDE database, the Inari FlowTriever and Penumbra Indigo systems were linked to 54 procedure-related deaths; pulmonary vascular perforation (48%) and cardiac injury (17%) were the most common causes (15). Even with good efficacy, these are serious risks—amplified in elderly patients. We recognize that smaller dedicated systems exist (e.g., Penumbra Indigo CAT 8F), but many hospitals, especially in resource-limited settings, do not have them. That makes a universally available alternative worthwhile.

The Guidezilla's 6F profile fits standard sheaths and standard technique, which should reduce vascular trauma without sacrificing clot removal. For elderly patients—especially those over 80 or with known vascular disease—smaller-bore catheters may tip the risk-benefit balance favorably.

Importantly, the rationale for pursuing catheter-directed thrombectomy in this elderly cohort is further supported by the suboptimal outcomes associated with conservative management alone. In intermediate-high risk PE, short-term mortality with anticoagulation alone has been reported to be up to 15% (14), a rate significantly higher than that observed in contemporary uncomplicated ST-segment elevation myocardial infarction cohorts undergoing primary percutaneous coronary intervention. Moreover, up to 23% of patients initially treated with anticoagulation alone may require secondary thrombolytic therapy within 24 h due to clinical deterioration (8), and a subset of intermediate-high risk patients will experience hemodynamic decompensation in the early hours following the acute event despite adequate anticoagulation (1). In elderly patients, the risks are further amplified by age-related physiological decline, multiple comorbidities, and increased bleeding susceptibility with systemic thrombolysis (16). A recent meta-analysis demonstrated that catheter-based therapies were associated with a significant 26% risk reduction in all-cause mortality compared to anticoagulation alone in intermediate-risk PE (20). These observations underscore that delayed or inadequate reperfusion in this vulnerable population carries substantial clinical consequences, and that a minimally invasive, small-bore catheter approach may offer a favorable balance between timely thrombus removal and bleeding avoidance when systemic thrombolysis is contraindicated.

One patient (Patient 4, 81-year-old woman) had complete clot clearance—Miller score went from 11 to 0 (100% clearance). This shows the Guidezilla can fully clear thrombus in selected elderly patients. Clearance varied widely across the group (61.1%–100.0%), probably because of differences in clot composition, burden, and distribution rather than any limitation of the catheter itself. Patient 5 had the highest pre-procedure Miller score (20) but a 70% clearance rate and the shortest hospital stay (4 days). That suggests a large clot load does not automatically mean a bad outcome. Patient selection matters, and in the right cases the Guidezilla may serve as a definitive treatment, not just a bridge.

Even at its small size, the Guidezilla cleared a mean 74.1% of clot burden—close to the 70.8%–83.1% reported for large-bore systems (14). Two things make this work: the inner lumen is relatively large (0.067 inches) for manual aspiration, and low-dose prourokinase (15–30 mg) can be injected directly into residual clot. This mechanical-plus-local-thrombolysis approach has been tested in ULTIMA, SEATTLE II, and OPTALYSE PE (9, 18, 19). Low-dose is key for elderly patients: systemic lysis carries >2% intracranial hemorrhage risk in those over 75 (1), often ruling it out. Our patients got only 15–30 mg—well under the 100 mg systemic dose—and none had major bleeding, including the two octogenarians.

All patients recovered quickly and meaningfully. Mean SpO₂ went from 84.5% ± 5.0% to 97.2% ± 0.8% in 24 h (*P* < 0.01). The patient who arrived in shock was off vasopressors within 24 h. Everyone survived to discharge; no recurrent PE or major events at 30 days. Mean stay was 7.8 ± 4.4 days, acceptable compared to published catheter-directed therapy data (14).

More evidence is building for catheter-directed PE therapy. Ismayl et al. found lower short-term mortality with catheter-directed thrombolysis than with anticoagulation alone (20). The FLASH registry reported a 1.8% major adverse event rate with the Inari FlowTriever (21). But these devices cost a lot and are often absent outside major centers, especially in developing countries (22, 23). The Guidezilla is already in most cath labs for coronary work. Our 74.1% clearance rate is close to dedicated systems, and our patients (mean age 75.8) are a group underrepresented in trials (2, 3).

Three practical points. First, when no dedicated PE catheter is available, the 6F Guidezilla can get the job done. Second, its small size suits elderly patients with fragile vessels and should lower complication rates. Third, any interventionalist with standard skills can do this—no specialized PE program needed. As the population ages and PE becomes more common (23), accessible techniques like this will matter more.

This study has several limitations. First, *n* = 6—hard to generalize. Second, retrospective and no control group, so we cannot directly compare against anticoagulation alone or large-bore devices. Third, incomplete data: post-procedure BNP in only three patients; no systematic echo assessment of RV function, so we cannot correlate thrombus reduction with RV improvement. Fourth, the Miller score is semi-quantitative and observer-dependent (13); we used two blinded reviewers to limit this. Fifth, bleeding risk was assessed qualitatively because the retrospective data lacked what is needed for HAS-BLED or IMPROVE scoring. Sixth, follow-up was only 30 days—longer-term outcomes are unknown.

Even with these limits, this series offers real-world data on an alternative for elderly patients who cannot get systemic thrombolysis. Prospective multicenter studies with more patients and longer follow-up would help confirm these results.

## Conclusion

The 6F Guidezilla catheter is a safe, effective option for thrombus aspiration and *in situ* thrombolysis in selected elderly patients with intermediate-high or high-risk PE—especially when systemic lysis is risky or vessels are fragile. In our series it cleared 74.1% of clot, similar to large-bore devices, with fast clinical recovery and no major bleeding. When dedicated catheters are not available or carry too much risk, this technique is a practical alternative.

## Data Availability

The raw data supporting the conclusions of this article will be made available by the authors, without undue reservation.
